# High body fat percentage is associated with primary aldosteronism: a cross-sectional study

**DOI:** 10.1186/s12902-020-00654-w

**Published:** 2020-11-23

**Authors:** Worapaka Manosroi, Pichitchai Atthakomol

**Affiliations:** 1 Division of Endocrinology, Department of Internal Medicine, Faculty of Medicine, Chiang Mai University Hospital, 110 Intrawarorot Road Soi 2, Si Phum, Amphoe Mueang Chiang Mai, Chiang Mai, 50200 Thailand; 2 Department of Orthopaedics, Faculty of Medicine, Chiang Mai University Hospital, Chiang Mai, 50200 Thailand

**Keywords:** Essential hypertension, Percent body fat, Primary aldosteronism

## Abstract

**Background:**

Excess aldosterone has been shown to be associated with obesity; however, there is currently a lack of data regarding the relationship between percentage of body fat and primary aldosteronism (PA), particularly pertaining to Asian populations. Furthermore, essential hypertension may mimic the condition of PA and there needs to be differentiation between the two. This study aimed to assess the association between percentage of body fat and PA.

**Methods:**

A cross-sectional study was conducted in the outpatient department of the Endocrine and Metabolism Unit of the tertiary care medical center in Thailand. Data was obtained from 79 patients who had been screened for PA due to hypertension in young-onset, hypokalemia, adrenal incidentaloma or resistance hypertension. Essential hypertension was defined as patients who had high blood pressure and were negative for PA screening. Body fat percentage was assessed by bioelectrical impedance analysis. The relationship between percentage of body fat and a diagnosis of PA was assessed using logistic regression analysis, including adjustment for confounding factors.

**Results:**

The participants were divided into a PA group (*n* = 41) and an essential hypertension group (*n* = 38). After controlling for confounding variables (age, sex, body mass index, cholesterol and insulin resistance status), the odds ratio of having PA in males with a percentage of body fat > 25% and females with percentage > 30% was 1.82 (95%CI = 1.79–1.86, *p* < 0.001).

**Conclusion:**

A higher percentage of body fat is associated with an increased risk of PA. Further studies need to be conducted to confirm the relationship between body fat percentage and PA.

## Background

Overweight and obesity have been reported to be strongly associated with hypertension [1]. A strong relationship between body mass index (BMI) and systolic blood pressure/diastolic blood pressure has also been demonstrated [2]. Obesity-related hypertension is considered to be one of the most common forms of hypertension, although the precise clarification is still inconclusive [3]. Previous studies have suggested that aldosterone may be the culprit in obesity-related hypertension [4, 5]. Aldosterone is a mineralocorticoid hormone mainly produced by the zona glomerulosa in the adrenal cortex. It is a key hormone for maintenance of sodium preservation systems in the kidney [6]. Elevated aldosterone levels have been observed in people with obesity, particulary abdominal obesity, and the level of aldosterone has been positively correlated with BMI [7]. In addition, an increased aldosteorne-renin ratio (ARR) has been described in obese hypertensive women although the exact explanation for this relationship is still unclear [5]. Various studies have suggested that adipose tissue is the source of adipokines, the so-called aldosterone-releasing factors (ARFs),which can stimulate aldosterone release from adrenal tissue [8]. The negative feedback regulation of ARFs in response to salt is impaired, especially in obese individuals, which can result in inappropriate secretion of aldosterone which may cause hypertension to occur [9].

The most common form of secondary hypertension is primary aldosteronism (PA) which has a reported prevalence in the general population of > 10% [10]. PA is a disorder in which aldosterone is autonomously produced and secreted independently of sodium status, angiotensin II and potassium status [11]. The prevalence of metabolic syndrome in PA patients is significantly higher than in patients with essential hypertension [12]. Links between higher visceral fat and greater subcutaneous fat percentage to metabolic syndrome have been reported [13]. One study demonstrated a moderate correlation between percentage of visceral fat and plasma aldosterone concentration in patients with idiopathic hyperaldosteronism which is one of the subtypes of PA [14]. However, as yet there are no conclusive data regarding the association between percentage body fat and PA in Asian populations, a lacuna that needs to be addressed [14, 15]. The commonly used cut-off points for body fat percentage used to define obesity in males and females are > 25 and > 30%, respectively [16, 17].

The established criteria for detection of possible PA is ratio between plasma aldosterone concentration (PAC) and plasma renin activity (PRA) known as the ARR. An ARR of more than 20 to 30 ng/dl per ng/(ml·h) is an indication of possible PA [18]. Aldosterone suppression can then be confirmed by the saline loading test, the oral sodium loading test or the fludocortisone suppression test [18]. Diagnosis of PA requires multiple complicated steps, and in some health care centers the screening tests necessary for the diagnosis may not be available. If specific characteristics, e.g., higher body fat percentage, could be shown to be associated with PA, it could potentially identify patients at high risk and hence those who may need further investigations for PA.

The objective of this study was to assess the association between percentage of body fat and primary aldosteronism in both men and women.

## Methods

This cross-sectional study was conducted at the outpatient Endocrine and Metabolism Unit of a tertiary medical center in the northern part of Thailand from September 2018–August 2019. The project protocol was approved by the Faculty of Medicine, Chiang Mai University Ethical Board (No.337/2018). We adhered to the Strengthening the Reporting of Observational Studies in Epidemiology (STROBE) Statement. Seventy-nine patients aged over 18 and suspected of having PA were included in this study. The indications for PA screening were based on the Endocrine Society Clinical Practice Guidelines which included hypertension in the young, hypokalemia, adrenal incidentaloma or resistance hypertension [18]. Written informed consent was obtained from all patients prior to their participation in the study. Exclusion criteria were: patients who had incomplete data for PAC, PRA levels and body composition; patients who used insulin, had chronic kidney disease (eGFR < 30 mL/min/1.73m^2^), Cushing’s syndrome, metastatic cancer, heart failure, or patients who used glucocorticoids.

### Diagnostic procedure for PA

Screening for PA was performed according to the Endocrine Society 2016 standard guidelines [18]. In addition, patients participating in the study who had hypertension stage 1 as defined in the American College of Cardiology/American Heart Association Task Force on Clinical Practice Guidelines 2017 (blood pressure ≥ 130/80 mmHg) and patients with other associated factors indicating potential PA were all screened for PA [19]. In brief, PAC and PRA were obtained between 0700 h and 0900 h [18]. The patients should have no hypokalemia (K should be > 3.5 mEq/L). Those who were currently taking beta-blockers, ACE inhibitors or ARBs were requested to discontinue these medications at least 2 weeks prior to the screening test. Patients using diuretics and mineralocorticoid receptor blockers were asked to discontinue those medications at least 4 weeks prior to the screening test. Only slow- release verapamil, hydralazine and/or α-blockers for hypertension control were allowed to be continued. Samples for PAC and PRA analysis were drawn from patients in the upright position after they had been seated for 5–15 min. Patients with an upright ARR greater than 20 ng/dl per ng/ml·h who also had a PAC of more than 15 ng/mL and who also had suppressed PRA underwent the normal saline suppression test for confirmation of PA. The confirmation test involved infusion of 500 mL of 0.9% NSS over 4 h while in the supine position. For those patients, PAC was measured after completion of the infusion; patients with PAC greater than 10 ng/mL were diagnosed as having PA [18].

### Anthropometric and body composition measurements

Weight was measured using a standardized digital scale with patients dressed in light clothing without shoes. A wall stadiometer was used to measure standing height of the patients without shoes. Weight and height were recorded to one decimal place. BMI was calculated as weight (kg)/height (m^2^). Waist circumference was measured midway between the lowest rib and the iliac crest in the horizontal plane with the patient at the end of a normal breath expiration.

A Tanita BC-545 N body composition analyzer (Tanita Cooperation, Tokyo, Japan) was used to assess bioelectrical impedance. Measuring current was standardized at 50 Hz; the age, sex and height of the patients were input manually. The patients were instructed to wear light clothing, stand barefoot and remain still while being measured. The percentage body fat was computed using the software installed in the machine. The contact electrode system was validated against dual-energy X-ray absorptiometry (DEXA) measurements with good correlation for fat mass [20].

### Assay methods

PAC was measured using direct ELISA assays (DiaMetra Ekit, Spello, Italy) with a reference range in the upright position of 3–40 ng/dL and in the supine position of 2–18 ng/dL. The intra-assay variability was < 9.7% and the inter-assay variability was < 11%. PRA was determined using direct ELISA assay (DRG Instruments GmBH, Germany) with a reference range of 0.06–4.69 ng/mL/hr. For assessment of insulin, electrochemiluminescence immunoassay (Electrosys Insulin, Cobas, Roche Diagnostics, Switzerland) was employed with reference values of 2.6–24.9 μU/mL and with intra- and inter-assay coefficients of variation for serum insulin of < 10%.

### Definitions

A high percentage body fat was defined as > 25% in males and > 30% in females [17]. A diagnosis of PA was defined as patients with screened ARR ≥20 ng/dl per ng/(ml·h) and a positive NSS infusion confirmation test [18, 21]. Essential hypertension was defined as patients who had high blood pressure, a reading of > 140/90 mmHg, and in whom the PA screening was negative. Other secondary causes of hypertension including pheochromocytoma, Cushing’s syndrome, renal artery stenosis or hyperthyroidism were excluded. Hypertension in young patients was defined as onset of hypertension in patients less than 40 years of age. Resistance hypertension was defined as patients with blood pressure > 140/90 mmHg while continuing three conventional antihypertensive medications including one diuretic or patients with controlled hypertension (blood pressure < 140/90 mmHg) while on four or more antihypertensive medications. Hypokalemia was defined as a history of serum potassium lower than 3.5 mEq/L whether diuretics were used or not. HOMA-IR (homeostatic model assessment for insulin resistance) was calculated according to the formula: fasting insulin (microU/L) x fasting glucose (nmol/L)/22.5.

#### Statistical analysis

Data were analyzed using STATA (Stata Corp., College Station,TX, USA). Categorical variables are presented as numbers and percentages, continuous variables as mean values and SDs. Comparison between categorical variables and PA was done using the Fisher’s exact test; in the case of continuous variables, the Student t-test was used for normally distributed variables and the Mann-Whitney U test for non-normally distributed variables. Multivariable logistic regression analysis was used to assess the association between percentage body fat and PA. The data are presented as crude and adjusted odds ratios (OR). The model was adjusted for age, BMI, serum cholesterol and HOMA-IR level and clustered by sex. Statistical significance value was set at *p* < 0.05.

## Results

### Baseline characteristics

Demographic, anthropometric and biochemical characteristics of the 79 patients are shown in Table 1**.** Data are categorized by results of PA screening which were PA and essential hypertension (negative result of PA screening). The mean age of the study subjects was 38.4 ± 14.5 years; 51% were male. Those with PA were significantly older than those without (*p* = 0.04). The majority of the males were in the group with PA. Average body fat percentage was higher in males than in females, but there was no significant difference in body fat percentage between the PA and essential hypertension groups (*p* = 0.214 in females and 0.941 in males). The most common indication for PA screening was hypertension diagnosed at age <40; the majority of screened patients were negative for PA. With the exception of serum triglycerides, there were no significant differences between groups in the case of any of the demographic, biochemical or anthropometric characteristics measured.

**Table 1:** Baseline characteristics

Characteristic	Total (***n*** = 79)	Primary aldosteronism (***n*** = 41)	Essential hypertension (***n*** = 38)	***P***-value
Age, years (mean ± SD)	38.4 ± 14.5	41.5 ± 15.2	35.0 ± 13.0	0.04
Male, n (%)	28 (35.4)	21 (51.2)	7 (18.4)	0.002
BMI, kg/m^2^(mean ± SD)	29.6 ± 6.8	30.0 ± 7.9	29.2 ± 5.2	0.60
Waist circumference, cm (mean ± SD)				
- Male	97.2 ± 13.1	96.9 ± 12.8	97.3 ± 13.4	0.90
- Female	91.1 ± 18.0	92.4 ± 19.6	86.7 ± 12.3	0.47
Smoking, n (%)	19 (24.1)	8 (19.5)	11 (28.9)	0.32
Underlying diseases, n (%)				
- Diabetes mellitus type2	9 (11.4)	7 (17.0)	2 (5.2)	0.09
- Dyslipidemia	20 (25.3)	11 (26.8)	9 (23.6)	0.74
- Cerebrovascular disease	6 (7.6)	4 (9.7)	2 (5.2)	0.45
- Coronary artery disease	2 (2.5)	1 (2.4)	1 (2.6)	0.95
Metabolic syndrome, n (%)	44 (55.7)	25 (60.9)	19 (50)	0.32
Indication for testing, n (%)				
- Hypertension in young (age <40)	44 (55.7)	16 (39.0)	28 (73.6)	0.002
- Hypertension with hypokalemia	5 (6.3)	4 (9.7)	1 (2.6)	0.19
- Adrenal incidentaloma	7 (8.9)	4 (9.7)	3 (7.8)	0.77
- Resistance hypertension	13 (16.5)	9 (21.9)	4 (10.5)	0.17
Plasma aldosterone concentration, ng/dL (mean ± SD)	58.1 ± 104.3	86.3 ± 138.1	24.3 ± 22.6	0.007
Plasma renin activity, ng/mL/hr., (mean ± SD)	3.2 ± 15.2	0.41 ± 0.4	6.09 ± 21.5	0.09
Serum potassium, mEq/L, (mean ± SD)	3.7 ± 0.6	3.6 ± 0.6	3.7 ± 0.5	0.38
Total cholesterol, mg/dL (mean ± SD)	180.8 ± 42.2	182.1 ± 39.3	179.3 ± 45.5	0.77
Triglycerides, mg/dL (mean ± SD)	146.5 ± 99.3	119.5 ± 49.3	174.8 ± 127.9	0.01
LDL, mg/dL (mean ± SD)	117.8 ± 42.9	120.6 ± 42.2	114.8 ± 43.9	0.55
HDL, mg/dL (mean ± SD)				
- Male	50.9 ± 24.1	54.3 ± 34.6	48.7 ± 14.1	0.42
- Female	55.2 ± 11.9	55.5 ± 11.6	54.2 ± 13.7	0.82
Fasting glucose, mg/dL (mean ± SD)	97.6 ± 21.4	97.7 ± 26.5	97.5 ± 14.6	0.96
Fasting insulin, μU/mL (mean ± SD)	21.9 ± 25.5	18.4 ± 16.6	25.6 ± 32.2	0.21
HOMA-IR (mean ± SD)	5.4 ± 6.5	4.7 ± 5.0	6.1 ± 7.7	0.31
Serum creatinine, mg/dL (mean ± SD)	1.1 ± 1.4	0.9 ± 0.5	1.2 ± 1.9	0.30
Body fat (%), n (%)				
- Male > 25	24 (85.7)	17 (41.4)	7 (18.4)	0.214
- Female > 30	15 (29.4)	6 (14.6)	9 (23.7)	0.941

*LDL* Low-density lipoprotein, *HDL* High-density lipoprotein, *HOMA-IR* Homeostatic model assessment of insulin resistance

### Association between percent body fat and a diagnosis of PA

The crude OR of a diagnosis of PA in males with a percentage body fat > 25% and females with > 30% was 1.75, 95%CI (1.28–2.39), *p* < 0.001. After adjusting for confounding variables (age, BMI, serum cholesterol and HOMA-IR) in the multivariable analysis, the OR of a PA diagnosis was 1.82, 95%CI (1.79–1.86), *p* < 0.001 (Table 2).

**Table 2:** Association of percent body fat and PA after adjusting for confounders and clustered by sex

Factor	Adjusted ORs	***P***-value	95% confidence interval
**Percent body fat** **> 25 (male)** **> 30 (female)**	1.82	< 0.001	1.79–1.86
Age^a^	1.04	< 0.001	1.03–1.05
BMI^a^	1.08	0.024	1.01–1.16
Serum cholesterol^a^	1.00	0.473	0.98–1.02
HOMA-IR^a^	0.93	0.274	0.82–1.05

*ORs* Odds ratio, *BMI* Body mass index, *HOMA-IR* Homeostatic model assessment of insulin resistance

^a^Confounders

## Discussion

This study highlights that a higher percentage body fat in hypertensive patients is significantly associated with an increase in risk of PA by a factor of 1.82. This finding suggests that physicians should consider screening for PA in hypertensives with a high percentage of body fat.

The study also identified some indirect evidence pertaining to the relationship between body fat percentage and the renin-angiotensin-aldosterone system. A prior study reported a correlation between PAC and obesity status in non-PA patients. In that study, PAC was found to be significantly higher in obese hypertensive than in non-obese normotensive patients [22]. Rossi et al. stated that in essential hypertension patients, BMI was related to PAC independent of age, sex and sodium intake status [23]. Another study reported that in obese patients a reduction of PAC was observed 3 months after bariatric surgery and that the reduction was associated with a decline in body weight, waist circumference and percent body fat [24]. Similarly, another study showed that a 5% reduction in body weight was associated with a 31% reduction in PAC [25]. Together, these results suggest a connection between PAC and obesity-related hypertension.

One study in PA patients reported a U-shaped correlation between PAC and BMI [26]. However, a study by Rossi et al. found no significant correlation between BMI and PAC [23]. Regarding the relationship between direct measurement of body fat percentage and PA, however, results were inconclusive. Shibayama et al. reported a significant correlation between percentage of visceral fat and PAC, but not between subcutaneous adipose area and PAC in PA patients with confirmed idiopathic hyperaldosteronism [14]. Another study, performed solely in females, reported that the subcutaneous fat area in obese female PA patients with idiopathic hyperaldosteronism subtype was higher than the same area in non-obese female patients with the syndrome [15]. This finding indicates a potential contribution of subcutaneous fat in the pathogenesis of PA in obese females.

The results of the present study suggest a possible interaction between adipose tissue and the production and/or secretion of aldosterone. A study in an animal model proposed that adipose tisuues contain ARFs which can stimulate aldosterone synthesis and secretion in adrenocortical cells [9]. On the other hand, the activation of mineralocorticoid receptors can also induce the differentiation of preadipocytes to mature adipocytes via the induction of cytokines [27]. Adipocyte-derived factors which can influence adrenal aldosterone secretion include leptin, adiponectin and complement-C1q TNF-related protein-1 (CTRP-1) [28]. It has also been found that increased leptin signaling can enhance CYP11B2, inducing increased aldosterone synthesis and release [29]. Moreover, adiponectin secreted by adipocytes can control aldosterone secretion via adiponectin receptors [30]. Similarly, CTRP-1 can increase intracellular Ca^2+^ levels and can lead to CYP11B2 expression which helps regulate adrenal aldosterone production [31].

In accordance with the Endocrine Society Clinical Practice Guidelines, obesity was not included as one of the indications for PA screening [18]. However, various reports have demonstrated that obesity is a key factor associated with PA, particularly in individuals with sleep apnea syndrome and/or metabolic syndrome [32, 33]. Body fat percentage has been shown to be linked to obesity and BMI [34]. However, in Asian populations, BMI measurements following WHO standards may underestimate body fat content [35]. In Asian people, direct measurement of body fat may be one of the indicators used to indicate obesity status. Patients who have high percentage of body fat may be considered for screening for PA.

Strengths of this study include that, unlike prior studies [14], this study used logistic regression analysis to enable adjustment for confounding factors (age, sex, BMI, serum cholesterol and HOMA-IR). These are factors which could potentially affect PA and body fat percentage determinations, making results both more interpretable and more accurate. Retrospective statistical power calculated for the primary objective was >80%.

This study does have some limitations. First, bioelectrical impedance was used to calculate body fat percentage. This method of measurement has not generally been considered the gold standard for measuring this value, so the results may not be as accurate in comparison with some other methods, e.g., dual-energy X-ray absorptiometry (DEXA). Additional validation is needed. Another limitation is that only members of the Thai population were included, so the results may not be representative of other Asian ethnic groups. Finally, the subtypes of PA (idiopathic hyperaldosteronsim and aldosterone producing adenoma) in this cohort were not determined by adrenal venous sampling due to resource limitation and some patients have chosen medical management over surgical management.

## Conclusion

There is a signficant relationship between higher body fat percentage (> 25% in males and > 30% in females) and PA. Physicians should be very aware of the potential for PA in those patients. Early screening tests, including for levels of plasma aldosterone and renin, should be performed, as delay in diagnosis can lead to negative outcomes which include congestive heart failure, myocardial infarction and chronic kidney disease.

## Data Availability

The datasets used in the analysis in this study are available from the corresponding author on reasonable request.

## Abbreviations

PA: Primary aldosteronism
BMI: Body mass index
ARR: Aldosterone-renin ratio
ARFs: Aldosterone releasing factors
ACE inhibitors: Angiotensin-converting enzyme inhibitors
ARBs: Angiotensin II receptor blockers
NSS: Normal Saline Solution
eGFR: Estimated glomerular filtration rate
STROBE: Strengthening the Reporting of Observational Studies in Epidemiology
PAC: Plasma aldosterone concentration
PRA: Plasma renin activity
DEXA: Dual-energy X-ray absorptiometry
ELISA: Enzyme-linked immunosorbent assay
HOMA-IR: homeostatic model assessment of insulin resistance
OR: Odds ratio
CI: Confidence Interval
